# Expression of endo-1, 4-beta-xylanase from *Trichoderma reesei *in *Pichia pastoris *and functional characterization of the produced enzyme

**DOI:** 10.1186/1472-6750-9-56

**Published:** 2009-06-16

**Authors:** Jun He, Bing Yu, Keying Zhang, Xuemei Ding, Daiwen Chen

**Affiliations:** 1 Institute of Animal Nutrition, Sichuan Agricultural University, Ya'an, Sichuan 625014, PR China; 2 Key Laborotary of Animal Disease-Resistance Nutrition, Ministry of Education, Beijing, PR China

## Abstract

**Background:**

In recent years, xylanases have attracted considerable research interest because of their potential in various industrial applications. The yeast *Pichia pastoris *can neither utilize nor degrade xylan, but it possesses many attributes that render it an attractive host for the expression and production of industrial enzymes.

**Results:**

The Xyn2 gene, which encodes the main *Trichoderma reesei *Rut C-30 endo-β-1, 4-xylanase was cloned into the pPICZαA vector and expressed in *Pichia pastoris*. The selected *P. pastoris *strains produced as 4,350 nkat/ml β-xylanase under the control of the methanol inducible alcohol oxidase 1 (*AOX1*) promoter. The secreted recombinant Xyn2 was estimated by SDS-PAGE to be 21 kDa. The activity of the recombinant Xyn2 was highest at 60°C and it was active over a broad range of pH (3.0–8.0) with maximal activity at pH 6.0. The enzyme was quite stable at 50°C and retained more than 94% of its activity after 30 mins incubation at this temperature. Using Birchwood xylan, the determined apparent *K*_m _and k_cat _values were 2.1 mg/ml and 219.2 S^-1^, respectively. The enzyme was highly specific towards xylan and analysis of xylan hydrolysis products confirmed as expected that the enzyme functions as endo-xylanase with xylotriose as the main hydrolysis products. The produced xylanase was practically free of cellulolytic activity.

**Conclusion:**

The *P. pastoris *expression system allows a high level expression of xylanases. Xylanase was the main protein species in the culture supernatant, and the functional tests indicated that even the non-purified enzyme shows highly specific xylanase activity that is free of cellulolytic side acitivities. Therefore, *P pastoris *is a very useful expression system when the goal is highly specific and large scale production of glycosyl hydrolases.

## Background

Xylans are major hemicellulose component of plant cell wall, usually accounting for 20%–30% of their total dry mass. As they are the second most abundant natural polysaccharide after cellulose, complete degradation of them could generate various forms of cellulosic biomass and allow utilization of such low-cost raw materials for industrial applications [1]. Xylans have a relatively complex structure based on a non-branched β-1,4-glycosidically linked xylose backbone. Depending on the origin, the backbone structure is substituted to various degrees with acetyl, L-arabinofuranosyl, glucuronyl and 4-*O*-methylglucuronyl groups [2]. Complete degradation of xylans requires the synergistic action of several enzymes of which EC3.2.1.8-endo-beta-1, 4-xylanases are the crucial enzymes for depolymerization [3]. In recent years, xylanses have attracted considerable research interest because of their potential industrial application, such as in biobleaching, paper making and in the food and animal feed industries [4-6]. Various microorganisms, such as bacteria, yeasts, and filamentous fungi were found to naturally secreted xylanases.

The *Trichoderma *species has long been shown to secrete large amounts of efficient xylan-degrading enzymes, which render it an attractive microorganism for industrial enzyme production. The two major endo-xylanases secreted by this fungus are Xyn1 and Xyn2. Xyn1 has an acidic pI (5.5), possesses a smaller, tighter groove than Xyn2, and a lower pH optimum [7]. Xyn2 has a basic pI (9.0) and a wider pH range. Both Xyn1 and Xyn2 produce similar hydrolysis end products. However, Xyn2 represents more than 50% of the total xylanolytic activity of this fungus and tends to produce larger oligosaccharides [5]. Today, the recombinant production hosts are preferred [8-10]. The recombinant production in fungal hosts, such as *Trichoderma reesei*, is not without problems, since they produce many enzymes at the same time. Although expression of some major enzymes (e.g. cellulase) has been knocked out, they still produce other enzymes that in certain applications can be problem [11].

Thus, production systems, in which a minimal amount of interfering side activities are produced, is desired. Xylanases can be produced in secreted form from *E. coli*, however, the production level is very low. [9,12-15]. The yeast *Pichia pastoris *can neither utilize nor degrade xylan, but it possesses many attributes that render it an attractive host for the expression and production of xylanases. As a eukaryote, *Pichia pastoris *has many of the advantages of higher eukaryotic expression systems such as protein processing, protein folding, and posttranslational modification, while being as easy to manipulate as *E. coli *or *Saccharomyces cerevisiae*. It is faster, easier, and less expensive to use than baculovirus or mammalian expression systems, and generally gives higher expression levels. As a yeast, it shares the advantages of molecular and genetic manipulations with *S. cerevisiae *and has the added advantage of 10 to 100 fold higher heterologous protein expression levels [16,17]. These features make *Pichia *very useful as a protein expression system.

In this study, we describe the molecular cloning of the *T. reesei *Xyn2 gene in *P. pastoris*. Expression of the Xyn2 gene in *P. pastoris *was obtained with the aid of multicopy plasmids, using the strong *P. pastoris *promoter-terminator expression cassettes derived from the inducible alcohol oxidase1 (*AOX1*) gene. In addition, the enzymatic properties of the recombinant xylanase were characterized.

## Results

### Cloning of the *T. reesei *Xyn2 gene

The mature Xyn2 gene was prepared from first-strand cDNA prepared from *T. reesei *Rut C-30 by using sequence-specific PCR primers. Thus a fragment of 570 bp of Xyn2 gene was obtained and sequenced [GenBank: EU532196]. The calculated molecular mass (21 kDa) is in good agreement with the molecular mass of native Xyn2 isolated from *T. reesei *[7]. The *T. reesei *Rut C-30 Xyn2 gene has the same nucleotide sequence with VTT-D-79125 [GenBank: S67287]. Both of them are 99% identical to wild type QM6α [GenBank: U24191], differing only two base pair substitutions (nucleotide 43, 272). The two substitutions probably resulted from its mutagenesis steps used in the mutant selection program [18,19].

### Expression of the recombinant Xyn2 in *P. pastoris*

**Figure 1:**
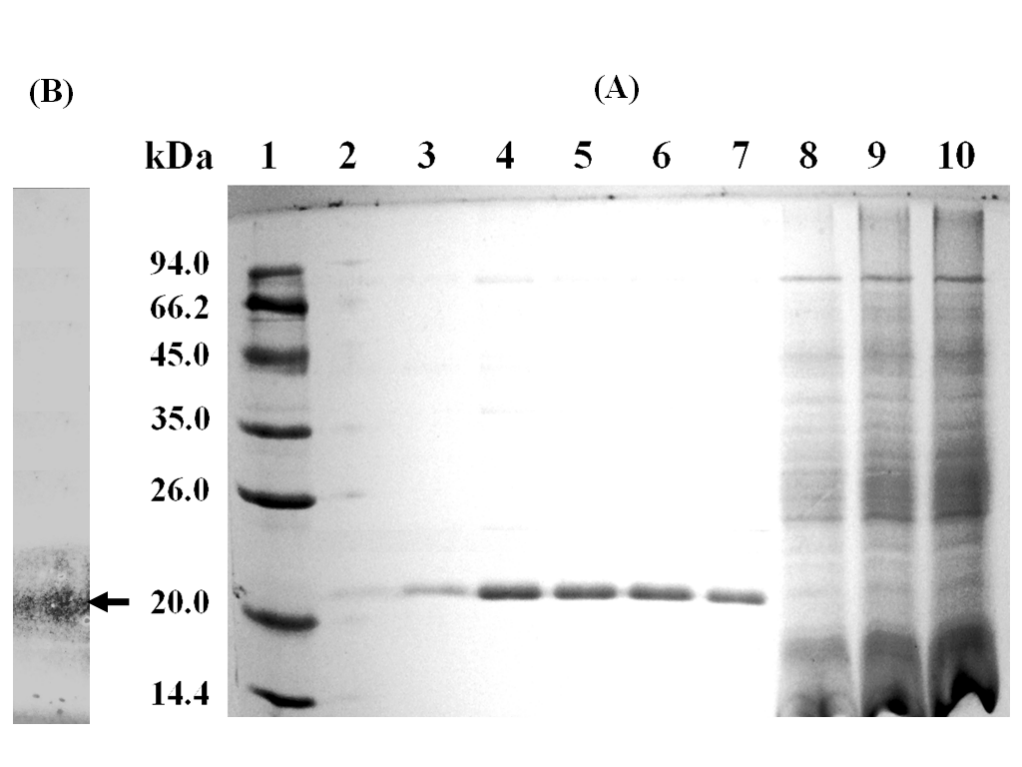
**SDS-PAGE and Zymogram analysis of expressed products**. (A) SDS-PAGE analysis for expressed products. Lane 1: protein marker; Lane 2–7: the culture supernatant containing recombinant xylanase from positive clones on day 1, 2, 3, 4, 5, and 6; Lane 8–10: cell lysate from recombinant *P. pastoris *on day 1, 3, and 5. (B) Zymogram analysis for xylanase activity.

The two specific primers (Up1 and Down1) were designated to incorporate the restriction enzyme sites *Eco*RI and *Not*I respectively at their 5' ends, allowing the directional cloning of the Xyn2 gene into pPICZαA expression vector. The target fragment was integrated into *P. pastoris *X-33 strain. The transformants were selected on YPDS plate containing zeocin and the integration of Xyn2 gene into AOX1 location in the genome was further confirmed by PCR. The most desired integrant was chosen for small-scale induction. The size of recombinant xylanase, determined by SDS-PAGE (Figure 1A) was 21 kDa, similar to that of the native xylanase secreted by *T. reesei*. SDS-PAGE also showed that the xylanase was the major protein (over 95% of total protein as detected by densitometer) secreted by *P. pastoris *into culture medium. Therefore, the procedure for protein purification was not necessary.

**Figure 2:**
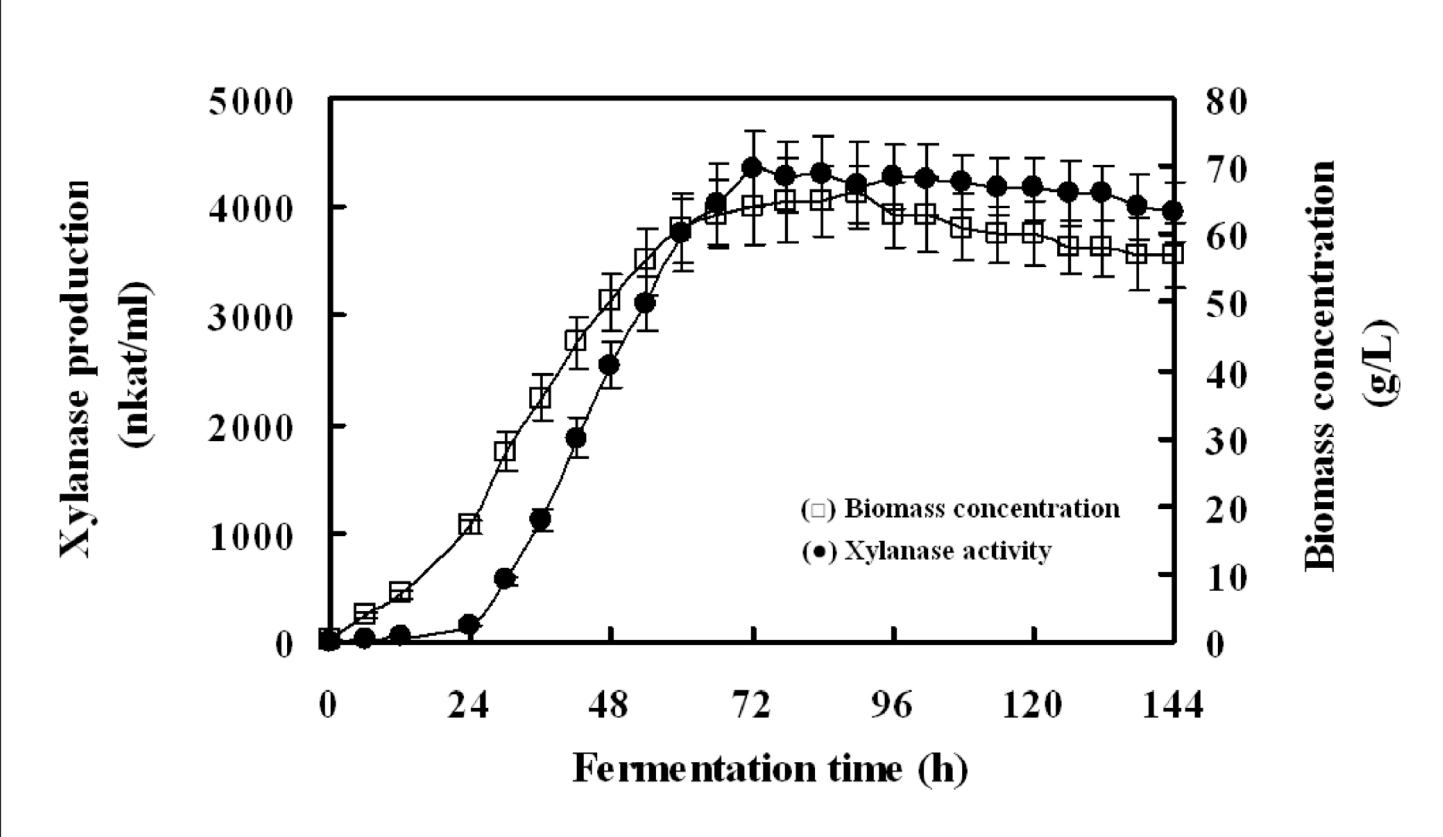
**Time course of xylanase activity produced by recombinant *P. pastoris***.

For optimal production of Xyn2, the recombinant *P. pastoris *was cultured in 2 L bioreactor. Both the highest xylanase activity (4,350 nkat/ml) and protein concentration (0.35 mg/ml) in culture supernatant was recorded after 72 h induction (Figure 2). However, the highest cell dry-weight (66 g/L) was obtained at 90 h. Both xylanase activity and cell dry-weight decreased slightly in the late induction period (after 96 h).

### Zymogram

When the culture medium (after induction) were subjected to native-PAGE, xylanolytic activity was detected using oat-spelt xylan as substrate; Immersion of the substrate gel in ethanol resulted in precipitation of undegraded xylan, which enhanced contrast and revealed only one clear band against a black background (Figure 1B).

### Effect of pH and temperature on β-xylanase activity

**Figure 3:**
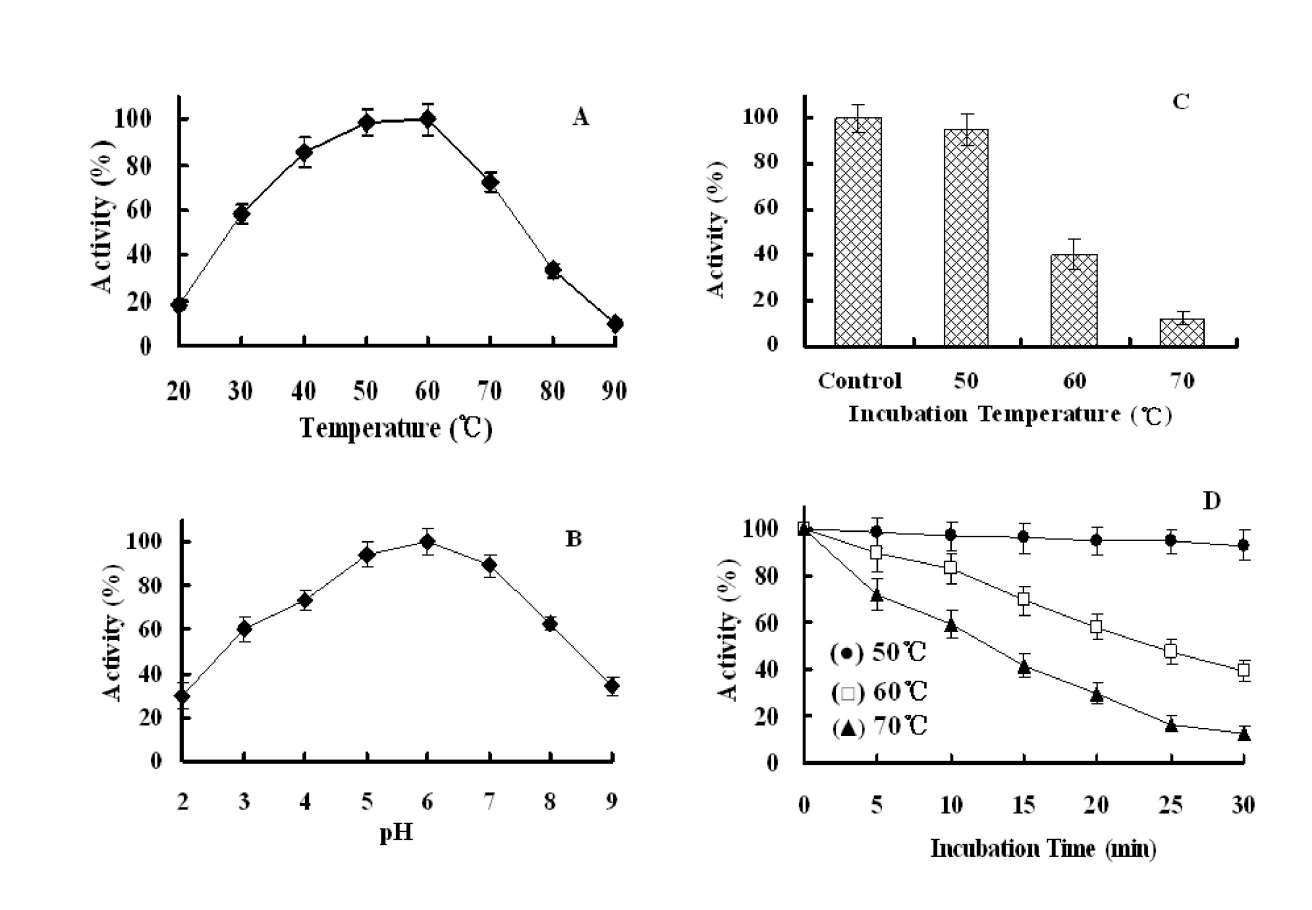
**Characterization of the recombinant Xyn2**. (A) Effect of temperature on the activity of Xyn2. (B) Effect of pH on the activity of Xyn2. (C) The temperature stability of Xyn2 was determined after preincubating the enzyme in the absence of the substrate for 30 min at 50, 60, and 70°C. (D) The thermostability of Xyn2 at different temperatures was determined by preincubating the enzyme at these temperatures in the absence of substrate for 5, 10, 15, 20, 25, and 30 min before measuring its activity. The xylanase activity prior to the preincubations at different temperature was taken as 100%.

Enzymatic assay at different temperatures revealed that the recombinant Xyn2 has an optimal activity at 60°C (Figure 3A). Activity decreased rapidly with temperature, i.e., at 80°C, activity of the recombinant protein represented only 30% of the optimum. Concerning the effect of the pH, the enzyme showed an optimal activity at approximately pH 6.0 (Figure 3B). When the pH was below 3.0 and above 8.0, only 30%–40% of the maximum activity was reached. Although the highest activity was measured at 60°C, the enzyme is not stable at this temperature (only 40% activity retained after 30 min incubation). However, the recombinant Xyn2 was stable at 50°C, and the total activity retained more than 94% after 30 min incubating at this temperature (Fig 3C, D).

### Substrate specificity and kinetic parameters

The hydrolytic activity of the recombinant enzyme on various substrates was determined (Table 1). The highest activity (108%) was observed with the Birchwood xylan followed by the oat-spelt xylan (100%). The enzyme exhibited low activities towards cellulosic substrates, such as Gellan gum (11%), Avicel (1.9%) and CMC (1.4%). The Michaelis-Menten constants were determined for the substrates. The *K*_m _and k_cat _were 2.1 mg/ml and 205.7 s^-1 ^for Birchwood xylan, and 1.8 mg/ml and 168.7 s^-1 ^for oat-spelt xylan, respectively.

**Table 1:** Substrate specificity and kinetic constants for the recombinant xylanase (All the values are the means of three replications)

**Substrate**	**Specific activity (nkat/ml)**	**Relative activity^1 ^(%)**	***K*_*m *_(mg/ml)**	**k_cat _(S^-1^)**
Oat-spelt xylan	4350 ± 125	100	1.8 ± 0.02	168.7 ± 11.6
Birchwood xylan	4687 ± 187	108	2.1 ± 0.11	205.7 ± 19.5
Beechwood xylan	3518 ± 114	83	1.4 ± 0.13	139.6 ± 12.1
Gellan gum	452 ± 25	11	NA^2^	NA
Avicel	78 ± 8	1.9	NA	NA
CMC	45 ± 4	1.4	NA	NA

^1^The activity for oat-spelt xylan was defined as 100%.

^2^Not available (The value is difficult to measure)

### Polysaccharide-binding properties

**Figure 4:**
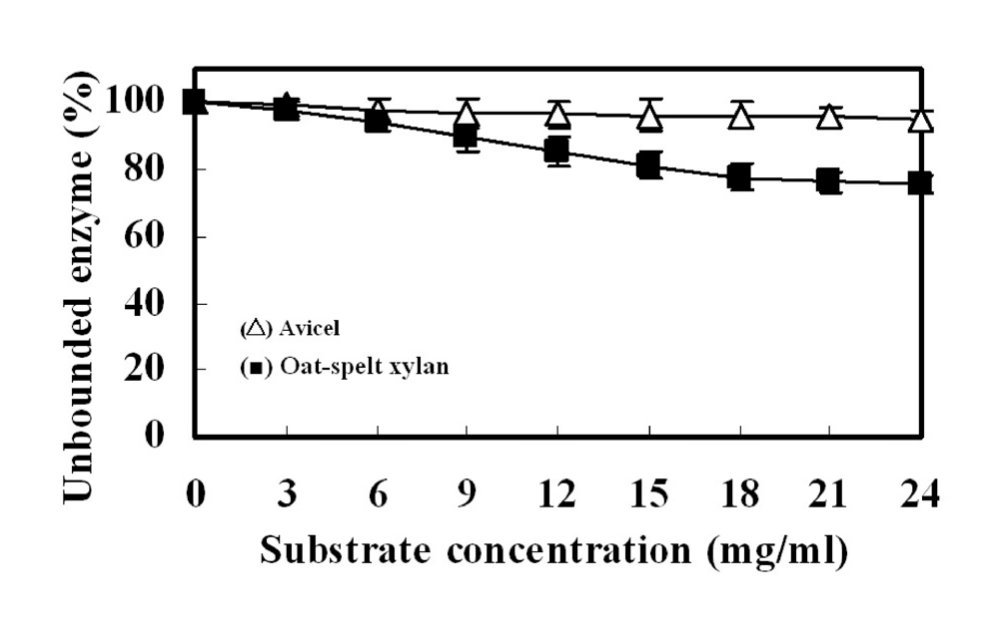
**Effect of different concentrations of Avicel and oat-spelt xylan on the binding ability of the recombinant Xyn2**. Xylanase (25 μg) was incubated with 1–24 mg/ml Avicel or oat-spelt xylan in 50 mM citrate phosphate (pH 5.0) at 4°C.

The polysaccharide-binding capacity of the recombinant Xyn2 was determined by incubating the enzyme with Avicel or oat-spelt xylan. As shown in Figure 4, the recombinant enzyme could not bind to Avicel, as about 95% enzyme activity still remained in supernatant. In contrast, the enzyme did show its capacity to bind to xylan and 75% of the enzyme activity remained in the supernatant.

### Enzymatic hydrolysis of xylans

**Figure 5:**
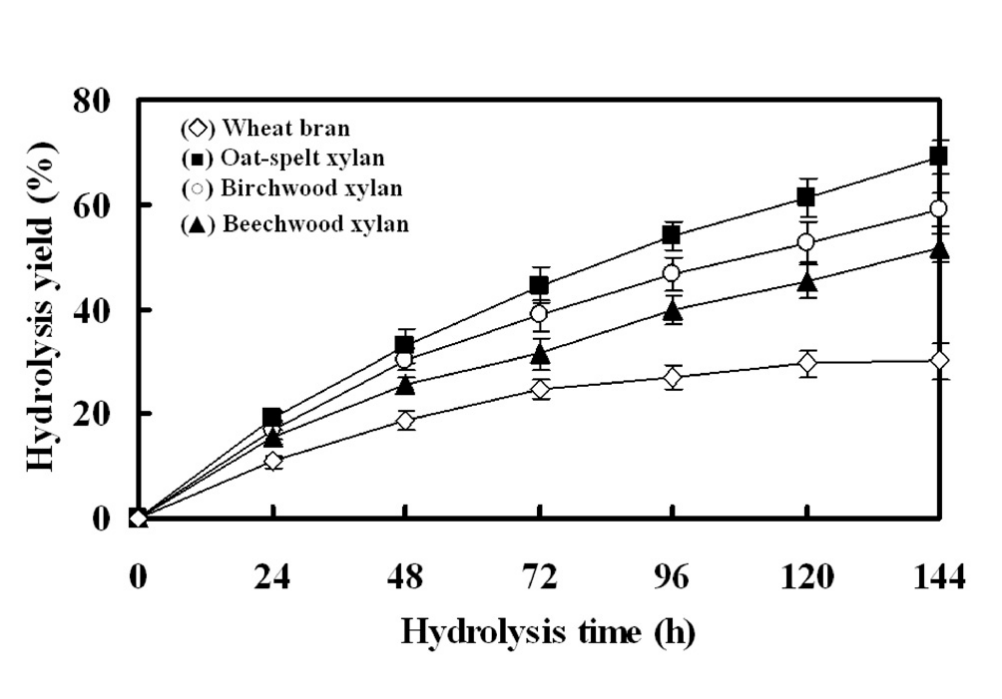
**Hydrolysis capacity of the recombinant Xyn2**. The hydrolysis yield of Oat-spelt xylan, Birchwood xylan, Beechwood xylan and Wheat bran using purified recombinant xylanase. Substrates (2.5 g) were incubated with 1000 nkat of the enzyme in 50 ml 50 mM citrate buffer (pH 5.0) and the reaction was carried out at 50°C with shaking at 150 rpm.

**Figure 6:**
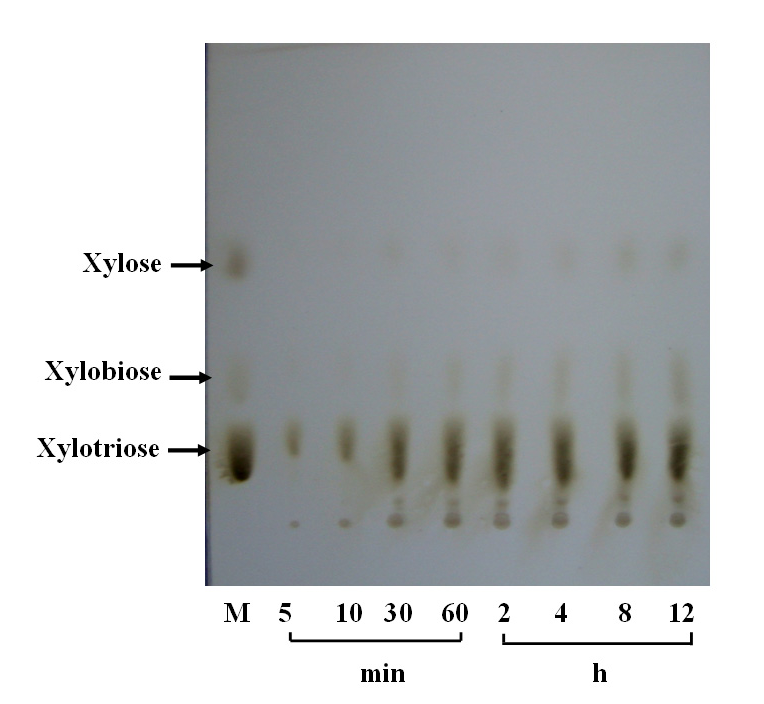
**Analysis of the hydrolyzed products by the recombinant Xyn2**. Oalt spelt xylan (40 mg) was incubated with 1000 nkat of the enzyme in 2 ml 50 mM citrate phosphate (pH 5.0) and the reaction was performed at 50°C for 12 h and then the hydrolyzates were analyzed by TLC.

The recombinant Xyn2 has been used to hydrolyze substrates at 5% concentration. Yield of enzymatic hydrolysis was highest when Birchwood xylan was used as the substrate (Figure 5). The wheat bran hydrolysis was lowest (only 30%), but the hydrolysis for both xylan and wheat bran linearly increased with the increasing of reaction time. The products of hydrolysis of oat-spelt xylan were analyased by TLC. The predominant hydrolysis end product of oat-spelt xylan was xylotriose (Figure 6). The xylotriose was produced within 5 min of the reaction period. As the reaction time increased, both the xylotriose and xylobiose concentration increased. These results confirmed that the recombinant Xyn2 was an endo-xylanase.

## Disccussion

The filamentous fungus *Trichoderma reesei *(also known as *Hypocrea jecorina*) is one of the most efficient xylanase and cellulase producers. The highest total beta-xylanase activity obtained in shake flasks for hyperproducing mutant *T. reesei *Rut C-30 was up to 5400 nkat/ml [20]. However, the *T. reesei *culture supernantant contains many enzymes involved in both hemicellulose and cellulose degradation, and these side acitivities can be a problem for use in many applications[21]. To resolve this problem, many researchers utilized heterologous expression system to produce xylanase from recombinant microorganisms [8-10]. In this study, the xyn2 gene from *T. reesei *Rut C-30 was successfully cloned and expressed in *P. pastoris*. For functional characterization, an unpurified enzyme was used and results indicated that even the non-purified enzyme shows highly specific xylanase activity. Therefore,*T. reesei *Xyn2 produced in *P. pastoris *has an advantage of showing xylanase activity that is practically free of harmful side activities.

Currently, the heterologous expression has become one of the main tools for the production of industrial enzymes [22]. And *P. pastoris *was one of the favorite expression hosts because of many advantages as we described previously[16,17]. The Xyn2 gene was inserted between a yeast promoter and transcription terminator on multicopy episomal plasmids to achieve high levels of gene expression [17]. For this purpose, we used the strong *P. pastoris AOX1 *promoter-terminator cassettes. In addition, the secretion signal sequence from *S. cerevisiae *α factor prepro peptide has been used to achieve a secretory expression. As a result, the recombinant xylanase was successfully secreted into the medium as a major protein with 95% purity and apparent molecular mass of 21 kDa. The highest total β-xylanase activity obtained in shake flasks for the most efficient recombinant *P. pastoris *strain was 4350 nkat/ml, which is much higher than we obtained from *E. coli *(445 nkat/ml) [12]. The expression level is also higher than that of levels obtained from recombinant *S. cerevisiae *strain (1,487 nkat/ml) or other *E. coli *expression systems [13-15]. Furthermore, the level may be higher than that of *T. reesei *Rut C-30 if one takes into account that the *T. reesei *culture supernatant contains other enzymes (i.e. xyn1 and β-xylosidases) involved in xylan degradation [8,20]. As shown in table 2 there are many family 11 xylanases that have been expressed in *P. pastoris *[1,23-27]. However, most of them showed quite low expression levels, except in one study a higher level (11052 nkat/ml) was obtained for Aspergillus niger xylanase with the purity of the recombinant protein being 86% [1]. As an unoptimized production level this is still missing from the efficiency of a recombinant *T. reesei *production system with published unoptimized production level of 32, 230 nkat/ml [28]. However, optimization is likely to further improve the production efficiency also in *P. pastoris*.

**Table 2:** Expression levels of different recombinant family 11 xylanases in *P. pastoris*

**Microorganisms**	**Host**	**Expression level^1^**	**Specific activity**	**Medium**	**Reference**
*T. reesei*	*P. pastoris*	261 U/ml	746 U/mg	BMMY	This study
*B. licheniformis*	*P. pastoris*	163.5 U/ml	122.9 U/mg	BMMY	[23]
*A. niger*	*P. pastoris*	NA^2^	175 U/mg	BMMY	[24]
*A. niger*	*P. pastoris*	NA	633 U/mg	BMMY	[25]
*T. lanuginosus*	*P. pastoris*	360 U/ml	NA	BMMY	[26]
*A. sulphureus*	*P. pastoris*	120 U/ml	NA	BMMY	[27]
*A. niger*	*P. pastoris*	663 U/ml	773.1 U/ml	BMMY	[1]

^1^1 U≈16.67 nkat

^2^Not available

In comparison to *Saccharomyces cerevisiae*, *P. pastoris *may have another advantage in the glycosylation of secreted proteins because it may not hyperglycosylate. The mature Xyn2 produced by *T. reesei *has a molecular mass of 21 kDa as deduced from the amino acid sequence. The molecular mass of *P. pastoris *Xyn2 determined by SDS-PAGE is almost the same. However, the molecular mass of the enzyme secreted by *S. cerevisiae *is 27 kDa [8]. There is thus a 6-kDa difference in the molecular mass of the Xyn2 secreted by *P. pastoris *and *S. cerevisiae*. This is caused by N-glycosylation [17], because *S. cerevisiae *tends to "hyperglycosylate" heterologous proteins. However, treatment of the *S. cerevisiae *Xyn2 with endoglycosidase F generated a new protein species with Molecular mass of 21 kDa, which corresponds to that of native Xyn2 produced by *T. reesei *[8]. Though this large, glycosylated protein was efficently secreted into the culture medium, the lower expression level (1,487 nkat/ml) was not suitable for a large scale production.

Because the enzyme was the predominant protein (over 95% of total protein) in the culture medium, the procedure for protein purification was not necessary. When Birchwood xylan was used as the substrate, the recombinant Xyn2 showed *K*_m _and k_cat _values of 2.1 mg/ml and 205.7 S^-1^, respectively. The pH and temperature optima of the recombinant Xyn2 compared well with the Xyn2 secreted by *S. cerevisiae *(Table 3). Both of them have a moderate thermostability and inactivated rapidly above 60°C[7,8]. However, the *P. pastoris *Xyn2 retained at least 40% of its total activity after 30 mins incubation at 60°C. The improved thermostability could be caused by amino acid mutation or glycosylation. In this study, the Xyn2 gene [GenBank: EU532196] was cloned from *T. reesei *Rut C-30. However, the Xyn2 gene expressed in *S. cerevisiae *was cloned from QM6α [GenBank: U24191]. There are two substitutions at nucleotides 43 and 272 of the Rut C-30 Xyn2 DNA sequence, resulting in two amino acids (Try14/Glu91) in the protein being different from those in the protein (His14/Gly91) encoded by the QM6α Xyn2 gene. In contrast, the *E. coli *Xyn2 seems to be more thermostable than *P. pastoris *and native Xyn2. Our laboratory has previously reported an *E. coli *expression of Xyn2 which presents a desired thermostability and retained at least 70% of its total activity after 30 mins incubation at 60°C. The improved thermostability may result from the N-terminal fusion peptide (51 amino acids). When the fusion peptide was deleted by enterokinase, the recombinant Xyn2 retained only 50% of activity (unpublished results). Like with *T. reesei *Xyn2 expressed in *S. cerevisiae*, the pH optimum was the same for Xyn2 expressed in *P. pastoris *(pH 6.0). The enzyme was active over the range of pH 3.0–8.0.

**Table 3:** Comparison of the molecular characteristics between native Xyn2 and recombinant Xyn2

	**Value for Xyn2 from**				
		
**Property**	***T. reesei***	***S. cerevisiae***	***P. pastoris***	***E. coli***	**Reference**
Expression level (nkat/ml)	5400^1^	1470	4350	445	[7,8,12]
Mol. mass (kDa)	20–21	27	21	24	[7,8,12]
Optimum pH	5	6	6	5	[7,8,12]
pH stability^2^	4.5–5.0	4.0–7.0	3.0–8.0	3.5–7.5	[7,8,12]
Optimum temp (°C)^3^	50–55	60	60	50	[5,8,12]
Temp. stability (30 min, 60°C)	NA^4^	NA	40^5^	70^5^	[7,8,12]

^1^*T. reesei *VTT-D-86271 (Rut C-30)

^2^More than 60% of maximal activity retained.

^3^Optimum temperature was assayed at temperatures ranging from 20°C to 90°C for 10 min.

^4^NA, not available.

^5^40% or 70% of total activity retained.

*T. reesei *Xyn2 is classified to family 11 xylanases and is characterized by its small molecular mass and alkaline isoelectric point [29]. The *T. reesei *Xyn2 have five binding sites (only three are found in Xyn1) for binding the xylopyranose rings in the vicinity of the catalytic site [30,31]. Only few family 11 xylanases are known to have substrate-binding domain, e.g. *Thermomonospora fusca *TfxA and *Streptomyces lividans *XylB [32,33]. TfxA binds to both cellulose and insoluble xylan, but the enzyme has activity against only xylan. In this study, the recombinant Xyn2 also exhibited a high activity for xylan, but a low activity for cellulosic substrates. These results were in good agreement with results from polysaccharide-binding analysis (Figure 4). When incubating the enzyme with Avicel, more than 95% unbound enzyme was detected in the supernatant.

The hydrolytic capacity of the recombinant Xyn2 on pretreated substrates was also determined. More than 60% hydrolysis was obtained when oat spelt xylan (5%) was used as the substrate, whereas less than 30% hydrolysis was obtained with wheat bran. The decreased hydrolysis could be due to the fact that wheat bran contains more cellulosic compounds than xylan. In addition, the wheat bran is more difficult to dissolve. Usually, family 11 xylanases hydrolyze xylan to form oligosaccharides of different lengths [34]. In this study, Xyn2 expressed in *P. pastoris *hydrolyzed the oat-spelt xylan predominantly to xylotriose and in smaller amounts to xylobiose and xylose (Figure 6), which confirmed the endo-acting nature of the recombinant Xyn2. The xylooligosaccharides (i.e. xylotriose) has been found to be helpful for the maintenance of a healthy intestinal microflora [9]. But, the production of xylooligosaccharides is remaining a time consuming and expensive process. Therefore, the recombinant Xyn2 may be used for the large-scale production of xylooligosaccharides.

## Conclusion

In this study, the *P. pastoris *expression system designed for the high-production of Xyn2 allows large quantities of enzyme to be obtained in shake flasks. In addition, the recombinant Xyn2 produced by *P. pastoris *are pure and completely free of any contaminating cellulases. The recombinant Xyn2 exhibited a high specificity and hydrolysis capacity towards xylan. Coupled with its broad pH profile, all these features make the enzyme very useful for various industrial applications, such as in the animal nutrition and feed science. These results also suggested that the *P. pastoris *expression system is more suibtable for producing family 11 xylanases than other heterologous expression system. Future research in our laboratory will be focused on the development of a more effective vector for xylanase expression in *P. pastoris *and the co-expression of β-xylosidase or genes coding debranching enzymes will also be considered.

## Methods

### Strains and media

*P. pastoris *X-33 was cultivated in YPD medium (1% yeast extract, 2% peptone, 2% glucose). *Trichoderma reesei *Rut C-30 was cultivated in basal medium (BM) [0.3% oat spelts xylan (Sigma), 0.4% KH_2_PO_4_, 1% (NH_4_)_2_HPO_4_, 1% peptone, 0.3% yeast extract] [35]. Both these organisms were cultured in 1 L flasks containing 100 to 200 ml of medium at 30°C on a rotary shaker at 150 rpm.

Recombinant plasmids were constructed and amplified in *Escherichia coli *DH5α cultivated at 37°C in Luria-Bertani liquid medium or Luria-Bertani agar. Ampicillin for selecting and propagating resistant bacteria was added to a final concentration of 100 μg/ml.

### RNA isolation

One liter of *T. reesei *Rut C-30 culture was incubated in oat spelts basal medium for 48 h at 30°C. The fungal mycelia were harvest by centrifugation and frozen under liquid nitrogen. The frozen mycelia were ground into a fine powder with a sterile mortar and pestle, and suspended in a mixture of Trizol reagent (Takara D312), and total cellular RNA was isolated as described by the manual.

### Gene amplification and sequencing

The first strand cDNA synthesis was carried out with 100 ng of total cellar RNA by using a two-step RT-PCR kit (Takara DRR019A) as specified by the supplier. The DNA fragment encoding the *T. reesei *Rut C-30 Xyn2 was isolated from a first-strand cDNA mix by PCR with the two oligonucleotides Up1 (5'-ATA**GAATTC**CAGACGATTCAGCCCGGCAC GGG-3') and Down1 (5'-TTA**GCGGCCGC**TTAGCT-GACGGTGATGGA AGCAGAGC-3') supplied with the *Eco *RI and *Not *I restriction sites respectively. These primers were based on the sequence of the *T. reesei *Xyn2 gene, as published by La Grange *et al *[8]. The PCR reaction was performed in 25-μl reaction mixtures (0.15 μM each primer, 1 μl of template DNA [about 10 ng of first-strand cDNA], 12.5 μl PCR premix [Boracker KT201–02]). Denaturation, annealing and polymerization were carried out for 1 min at 94°C, 1 min at 58°C, and 1 min at 72°C, respectively for 35 cycles. The PCR product was directly cloned to the pMD18T Simple Vector (TaKaRa D101A) and sequenced by Invitrogen (Shanghai, China) Co., Lid. The sequence was analyzed using the software package DNAman 5.0 (Lynnon Biosoft, USA) and the homology was analyzed in GenBank with the BLAST programs .

### Construction and transformation of the recombinant plasmid

An *E. coli*/*P. pastoris *shuttle vector, pPICZαA (Invitrogen), was used to achieve secreted expression of xylanase. The mature Xyn2 gene obtained from PCR amplification was gel-purified and digested with *Eco*RI and *Not*I (Takara, Dalian) before cloning into pPICZαA. After transformed into *E. coli *DH5α, recombinant plasmids were selected on Luria-Bertani agar containing 25 μg/ml zeocin. The selection was checked by restriction analysis and sequencing.

For *P. pastoris *integration, about 10 μg of recombinant plasmid was linearized with *sac*I, and transformed in *P. pastoris *by electroporation methods as described by the manufacturer (Bio-Rad, USA). The transformants were selected at 28°C on the YPDS agar plates containing 100 μg/ml zeocin. The integration of the Xyn2 gene into the genome of *P. pastoris *was confirmed by PCR using 5'AOX1 and 3'AOX1 primers.

### Expression of recombinant xylanase in *P. pastoris*

*P. pastoris *transformants were grown in 20 ml of fresh buffered minimal glycerol complex medium, BMGY [1% yeast extract, 2% peptone, 100 mM potassium phosphatate (pH 6.0), 1.34% YNB, 0.0004% biotin, and 1% glycerol] at 30°C until an OD_600 _of 5~6 was reached.

Then, the cell pellet was harvested and resuspended in 100 ml buffered minimal methanol medium, BMMY [1% yeast extract, 2% peptone, 100 mM potassium phosphatate (pH 6.0), 1.34% YNB, 0.0004% biotin, and 0.5% methanol]. Sufficient supply of oxygen was assured by cultivation of the recombinant *P. pastoris *in 1 L flask (1:10 culture per flask volume ratio) at 250 rpm agitation throughout induction period. Absolute methanol was added every 24 h to a final concentration of 1% to maintain induction. The culture supernatant was collected every day by centrifugation. The supernatant was stored at -80°C before SDS-PAGE and analysis of its biochemical properties.

### Optimization of Xyn2 production in a 2 L bioreactor

A large scale production was performed in a 2 L bioreactor (B. Braun Sartorius Ltd.). Recombinant *P. pastoris *was grown in 200 ml BMGY medium. Temperature and pH were maintained at 30°C and 6.0, respectively throughout growth phase for 16–18 h. Agitation was kept within the range of 300–400 rpm. To induce enzyme production, cell pellets were resuspended in 800 ml of BMMY medium containing 1% methanol. The cultivation was maintained for 6 days by adding absolute methanol to a final concentration of 1% every day. Relative percentage of dissolved oxygen was maintained above 30% via adjusting agitation rate. Supernatants were collected every day and kept at -80°C before analysis. Cell pellets were washed, and dried at 80°C until constant weight was achieved.

### SDS-PAGE

Sodium dodecyl sulfate-polyacrylamide gel electrophoresis (SDS-PAGE) on 15% polyacrylamide was performed by the method of Laemmli [36]. The protein fractions were boiled for 3 min and applied to the gel. Proteins were visualized by Coomassie brilliant blue R 250 staining. The protein concentration was determined by the Bradford assay using bovine serum albumin as a standard [37].

### Zymogram analysis

The recombinant protein exhibiting xylanolytic activity was detected by running it on native-PAGE (All denaturants were removed from SDS-PAGE) as described by Royer and Nakas [38]. The gel was washed twice with distilled water and overlaid on substrate gel (1.5% oat spelt xylan, 1.5% agar in 50 mM sodium phosphate buffer, pH 5.0). The gels were next smoothed to remove bubbles, wrapped in plastic, and incubated at 50°C for 20 min. The gels were finally separated, and the substrate gels containing oat spelt xylan were immersed in 95% ethanol for 45 min and were photographed while raised approximately 12 in. (ca. 30 cm) above a black background.

### Enzyme activity assays

Xylanase activity was assayed by the method described by Bailey *et al*. [39], with 1% birchwood xylan xylan (Sigma) as the substrate at 50°C for 10 min. Appropriate dilutions of the recombinant protein (culture supernatant) in 50 mM sodium citrate buffer (pH 5.0) were used as the enzyme source. The amount of released sugar was determined by the dinitrosalicylic acid method described by Miller *et al*. [40]. One unit (nkat) of xylanase activity was defined as the quantity of enzyme that liberated reducing sugar at the rate of 1 nanomole per second. The temperature optimum was measured by performing the xylanase activity assay at temperatures ranging from 20°C to 90°C. Thermostability was tested by heating enzyme samples for different times at various temperatures, and the activity was assayed at 50°C for 10 min. Assays at different pH values were performed at the optimal temperature over a pH range of 3.0 to 8.0. The buffer used were 50 mM citrate (pH 3.0), 50 mM citrate phosphate (pH 4.0 to 7.0), and 50 mM phosphate (pH 8.0), respectively.

### Substrate specificity and kinetic parameters

Substrate specificity of the enzyme was determined using different cellulose and hemicellulose substrates. The reaction was carried out in 50 mM citrate phosphate (pH 5.0) containing 2.0 mg/ml of each substrate at 50°C for 10 min. For each assay, six different substrate concentrations were prepared in 50 mM citrate phosphate (pH 5.0), and incubated with the purified enzyme at 50°C for 5 min. The *K*_m _and k_cat _values were calculated from the kinetics data as described by Jiang *et al *[9].

### Polysaccharide-binding properties

The polysaccharide-binding capacity was determined by the method described by Tenkanen *et al *[41]. Purified Xyn2 (30 μg) was incubated with different concentrations of Avicel or oat-spelt xylan in 50 mM citrate phosphate buffer (pH 5.0), at 4°C for 1 h with slow shaking. After centrifugation (10000 × g, 5 min), the supernatant was collected and tested for its xylanase activity. Unbound enzyme was determined by measuring residual activity in the supernatant.

### Analysis of hydrolytic capacity and products

The hydrolysis of xylan-based substrates was carried out in 100 ml conical flask containing 50 ml citrate buffer (pH 5.0, 50 mM), 2.5 g xylan or wheat bran, 5 mg sodium azide and enzyme preparation (1000 nkat) as described by Adsul *et al *[42]. The hydrolysis was performed for 6 days at 50°C, with a stirring rate of 150 rpm. The samples were analyzed for the reducing sugars after suitable time intervals. The hydrolyzed products of xylan was analyzed by the thin-layer chromatography (TLC) using silica gel plates 60 F 254 (E. Merck, Germany). Aliquots (100 μl) of the samples were collected at 5, 10, 30 min, 1, 2, 4, 8, and 12 h of the incubation period and 1 μl of the aliquot was spotted on the TCL plates. The plates were subsequently developed with two runs of acetonitrile-water (85:15, v/v) followed by heating for a few minutes at 130°C in an oven after spraying the plates with a methanol-sulfuric acid mixture (95:5, v/v) [9]. A xylooligosaccharide mixture (Suntory Ltd, Japan) consisting of xylose, xylobiose, and xylotriose was used as the standard.

## Authors' contributions

HJ and YB participated in the experimental design, carried out the molecular genetic and biochemical experiments, participated in data interpretation and helped draft the manuscript. ZK and DX conceived the study. CD directly supervised the project, participated in its experimental design and data interpretation and was responsible for writing the manuscript. All authors have read and approved the manuscript.

## References

[B1] Ruanglek V, Sriprang R, Ratanaphan N, Tirawongsaroj P, Chantasigh D, Tanapongpipat S, Pootanakit K, Eurwilaichitr L (2007). Cloning, expression, characterization, and high cell-density production of recombinant endo-1, 4-β-xylanase from *Aspergillus niger *in *Pichia pastoris*. Enzyme Microb Technol.

[B2] Coughlan MP, Hazlewood GP (1993). β-1,4-D-Xylan-degrading enzyme systems: biochemistry, molecular biology and applications. Biotechnol Appl Biochem.

[B3] Beg QK, Kapoor M, Mahajan L, Hoondal GS (2001). Microbial xylanases and their industrial applications: a review. Appl Microbiol Biotechnol.

[B4] Buchert J, Ranua M, Kantelinen A, Viikari L (1992). The role of two *Trichoderma reesei *xylanases in bleaching of pine kraft pulp. Appl Microbiol Biotechnol.

[B5] Tenkanen M, Puls J, Potanen K (1992). Two major xylanases of *Trichoderma reesei*. Enzyme Microb Technol.

[B6] Suchita N, Ramesh CK (2006). Bleaching of wheat straw-rich soda pulp with xylanase from a thermoalkalophilic *Streptomyces cyaneus *SN32. Bioresource Technol.

[B7] Törrönen A, Mach RL, Messner R, Gonzalez R, Kalkkinen N, Harkki A, Kubicek CP (1992). The two major xylanases from *Trichoderma reesei *: characterization of both enzymes and genes. Biotechnology.

[B8] La Grange DC, Pretorius IS, Van Zyl WH (1996). Expression of a *Trichoderma reesei *β-xylanase gene (XYN2) in *Saccharomyces cerevisiae*. Appl Environ Microbiol.

[B9] Jiang ZQ, Deng W, Zhu YP, Li LT, Sheng YJ, Hayashi K (2004). The recombinant xylanase B of *Thermotoga maritima *is highly xylan specific and produces exclusively xylobiose from xylans, a unique character for industrial applications. Journal of Molecular Catalysis B: Enzymatic.

[B10] Saarelainen R, Paloheimo M, Fagerström R, Suominen PL, Nevalainen KMH (1993). Cloning, sequencing and enhanced expression of the Trichoderma reesei endoxylanase II (pI9) gene. Mol Gen Genetics.

[B11] Paloheimo M, Mantyla A, Kallio J, Puranen T, Suominen P (1997). Increased production of xylanase by expression of a truncated version of the *xyn11*A gene from *Nonomuraea flexuosa *in *Trichoderma reesei*. Appl Environ Microbiol.

[B12] Jun H, Keying Z, Bing Y, Xuemei Ding, Daiwen Chen (2009). Expression of a *Trichoderma reesei *β-xylanase gene in *Escherichia coli *and the activity of the enzyme on fiber-bond substrates. Protein Expr Purif.

[B13] Chenyan Z, Jiangyu B, Shanshan D, Jin W, Jie Z, Minchen W, Wu W (2008). Cloning of a xylanase gene from *Aspergillus usamii *and its expression in *Escherichia coli*. Bioresource Technol.

[B14] Junli H, Guixue W, Li X (2006). Cloning, sequencing and expression of the xylanase gene from a *Bacillus subtilis *strain B10 in *Escherichia coli*. Bioresource Technol.

[B15] Yang RobertCA, Roger MACKenzie C, Bilous Doris, Seligy VernerL, Narang SaranA (1988). Molecular cloning and expression of a xylanase gene from *bacillus polymyxa *in *Escherichia coli*. Appl Environ Microbiol.

[B16] Buckholz RG, Gleeson MAG (1991). Yeast Systems for the Commercial Production of Heterologous Protein. Biotechnology.

[B17] Romanos MA, Scorer CA, Clare JJ (1992). Foreign Gene Expression in Yeast: A Review. Yeast.

[B18] Bailey MJ, Nevalainen LMH (1981). Induction, isolation and testing of stable *Trichoderma reesei *mutants with improved production of solubilizing cellulose. Enzyme Microb Technol.

[B19] Sheir-Neiss G, Montenecourt BS (1984). Characterization of the secreted cellulases of *Trichoderma reesei *wild type and mutants during controlled fermentations. Appl Mcrobiol Biotechnol.

[B20] Bailey MJ, Buchert J, Viikari L (1993). Effect of pH on production of xylanase by *Trichoderma reesei *on xylan- and cellulose-based media. Appl Microbiol Biotechnol.

[B21] Wong KKY, Saddler JN (1992). *Trichoderma *xylanases, their properties and application. Critical Reviews in Biotechnology.

[B22] Kirk O, Borchert TV, Fuglsang CC (2002). Industrial enzyme application. Curr Opin Biotechnol.

[B23] Liu MQ, Liu GF (2008). Expression of recombinant *Bacillus licheniformis *xylanse A in *Pichia pastoris *and xylooligosaccharides released from xylans by it. Protein Expession and Purification.

[B24] Liu MQ, Weng XY, Sun JY (2006). Expression of recombinant *Aspergillus niger *xylanse A in *Pichia pastoris *and its action on xylan. Protein Expression and Purification.

[B25] Sun JY, Liu MQ, Xu YL, Xun ZR, Pan L, Cao H (2005). Improvement of the thermostability and catalytic activity of a *mesophilic *family 11 xylanase by N-terminus replacement. Protein Expression and Purification.

[B26] Damaso MCT, Almeida MS, Kurtenbach E, Martins OB, Pereira N, Andrade CMMC, Albano RM (2003). Optimized expression of a thermostable xylanase from *Thermomyces lanuginosus *in *Pichia pastoris*. Appl Environ Microbiol.

[B27] Cao Y, Qiao J, Li Y, Lu W (2007). De novo synthesis, constitutive expression of *Aspergillus sulphureus *beta-xylanase gene in *Pichia pastoris *and partical enzymic characterization. Appl Microbiol Biotechnol.

[B28] Paloheimo M, Mantyla A, Kallio J, Puranen T, Suominen P (2007). Increased production of xylanase by expression of a truncated version of the xyn11A gene from Nonomuraea flexuosa in *Trichoderma reesei*. Appl Environ Microbiol.

[B29] Janis J, Rouvinen J, Leisola M, Turunen O, Vainiotalo P (2001). Thermostability of endo-1, 4-β-xylanase II from *Trichoderma reesei *sudied by electrospary ionization Fourier-transform ion cyclotron resonance MS, hydrogen/deuterium-exchange reactions and dynamic light scattering. Biochem J.

[B30] Jeffries TW (1996). Biochemistry and genetics of microbial xylanases. Environ Biotechnol.

[B31] Davies G, Henrissat B (1995). Structures and mechanisms of glycosylhydrolases. Structure.

[B32] Black GW, Hazlewood GP, Millwardsadler SJ, Laurie JI, Gilbert HJ (1995). A modular xylanase containing a novel noncatalyutic xylan-specific binding domain. Biochem J.

[B33] Irwin D, Jung ED, Wilson DB (1994). Characterization and sequence of a *Thermomonospora fusca *xylanase. Appl Environ Microbiol.

[B34] Biely P (1985). Microbial xylanolytic systems. Trends Biotechnol.

[B35] Matsuo M, Yasui T (1984). Purification and some properties of β-xylanase from *Trichoderma viride*. Agric Biol Chem.

[B36] Laemmli UK (1970). Cleavage of structural proteins during the assembly of the head of bacteriophage T4. Nature (London).

[B37] Bradford MM (1976). A rapid sensitive method for the quantitation of microgram quantities of protein utilizing the principle of protein-dye binding. Anal Biochem.

[B38] Royer JC, Nakas JP (1990). Simple, sensitive zymogram technique for detection of xylanase activity in polyacrylamide gels. Appl Environ Microbiol.

[B39] MJ Bailey, Biely P, Poutanen K (1992). Interlaboratory testing of methods for assay of xylanase activity. J Biotechnol.

[B40] Miller GL, Blum R, Glennon WE, Burton AL (1960). Measurement of carboxymethylcellulase activity. Anal Biochem.

[B41] Tenkanen M, Buchert J, Uiikari L (1995). Binding of hemicellulases on isolated polysaccharide substrates. Enzyme Microbe Technol.

[B42] Adsul MG, Bastawde KB, Varma AJ, Gokhale DV (2007). Strain improvement of *Penicillium janthinellum *NCIM1171 for increased cellulase production. Bioresource Technol.

